# SVM-Based Prediction of Propeptide Cleavage Sites in Spider Toxins Identifies Toxin Innovation in an Australian Tarantula

**DOI:** 10.1371/journal.pone.0066279

**Published:** 2013-07-22

**Authors:** Emily S. W. Wong, Margaret C. Hardy, David Wood, Timothy Bailey, Glenn F. King

**Affiliations:** 1 Division of Chemistry and Structural Biology, Institute for Molecular Bioscience, The University of Queensland, St Lucia, Australia; 2 Division of Genomics and Computational Biology, Institute for Molecular Bioscience, The University of Queensland, St Lucia, Australia; Swiss Institute of Bioinformatics, Switzerland

## Abstract

Spider neurotoxins are commonly used as pharmacological tools and are a popular source of novel compounds with therapeutic and agrochemical potential. Since venom peptides are inherently toxic, the host spider must employ strategies to avoid adverse effects prior to venom use. It is partly for this reason that most spider toxins encode a protective proregion that upon enzymatic cleavage is excised from the mature peptide. In order to identify the mature toxin sequence directly from toxin transcripts, without resorting to protein sequencing, the propeptide cleavage site in the toxin precursor must be predicted bioinformatically. We evaluated different machine learning strategies (support vector machines, hidden Markov model and decision tree) and developed an algorithm (SpiderP) for prediction of propeptide cleavage sites in spider toxins. Our strategy uses a support vector machine (SVM) framework that combines both local and global sequence information. Our method is superior or comparable to current tools for prediction of propeptide sequences in spider toxins. Evaluation of the SVM method on an independent test set of known toxin sequences yielded 96% sensitivity and 100% specificity. Furthermore, we sequenced five novel peptides (not used to train the final predictor) from the venom of the Australian tarantula *Selenotypus plumipes* to test the accuracy of the predictor and found 80% sensitivity and 99.6% 8-mer specificity. Finally, we used the predictor together with homology information to predict and characterize seven groups of novel toxins from the deeply sequenced venom gland transcriptome of *S. plumipes*, which revealed structural complexity and innovations in the evolution of the toxins. The precursor prediction tool (SpiderP) is freely available on ArachnoServer (http://www.arachnoserver.org/spiderP.html), a web portal to a comprehensive relational database of spider toxins. All training data, test data, and scripts used are available from the SpiderP website.

## Introduction

**Figure 1:**
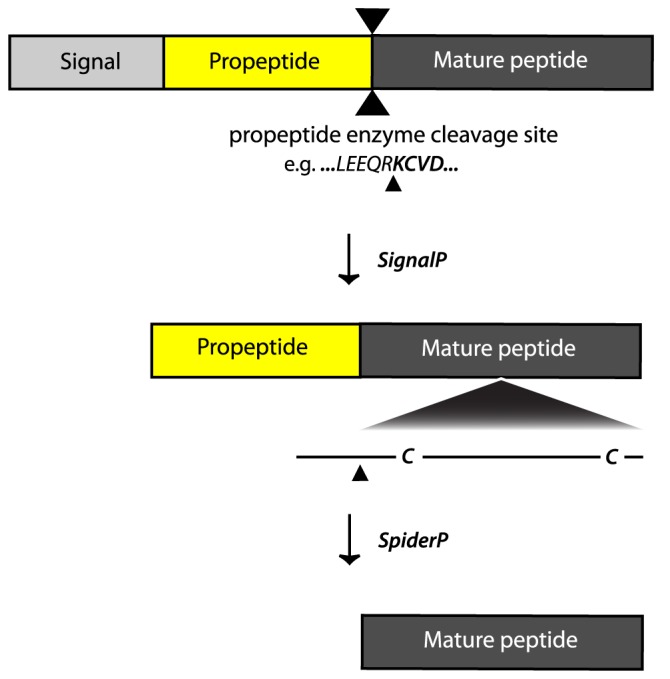
Bioinformatic prediction of signal and propeptide cleavage sites in spider toxin prepropeptide precursors using a combination of SignalP [10] and SpiderP. Propeptide convertases recognizes the propeptide cleavage site, which is typically located N-terminal to a cysteine-rich scaffold.

Spiders are the dominant insect killers and the most successful venomous animal on the planet. Their evolutionary success is due in large part to the evolution of exceedingly complex venoms [1], which have been predicted to contain as many as 10 million unique peptides [2]. Most spider-venom peptides are produced as prepropeptide precursors containing N-terminal signal peptide and propeptide regions in addition to the C-terminal region that will become the mature toxin [3]. Proteolytic enzymes excise the propeptide and signal peptide in order to release the active toxin (Fig. 1). Mature spider-venom peptides tend to be cysteine-rich, and the propeptide is always N-terminal to the cysteine-rich scaffold which restricts the access of proteolytic enzymes [4].

Propeptide regions allow spatial and temporal control of toxin activation, providing a means by which the peptide can be safely shuttled to its final destination. Thus, the propeptide can prevent aberrant activation of the toxin, which might have lethal consequences for the spider. Moreover, propeptides can facilitate protein folding [5] and encode certain types of posttranslational modifications [6]. In order to investigate the evolutionary trajectory of spider toxins and study toxin structure and function, it is necessary to identify the mature region in precursor toxin sequences. Given the enormous number of spider toxins [2], the experimental approach requiring fractionating whole venom followed by sequencing of amino acids is highly time-consuming and expensive, motivating the development of an *in silico* strategy.

Propeptide cleavage is catalysed by a different set of enzymes to those involved in removal of the signal peptide during protein transfer across the endoplasmic reticulum and through the secretory pathway. Propeptide convertases (PPCs) constitute the major family of endoproteases responsible for cleaving propeptides from prohormones and neuropeptides [4]. In eukaryotes, a large family of PPC typically recognizes a dibasic motif (usually ‘KK’, ‘RK’ or ‘KR’) on a protein substrate, and by cleaving at the C-terminal end of the motif, releases the active peptide from the proregion. Other types of proteases can recognize the same cleavage signals. For example, Tex31, a cysteine-rich secreted protease from cone snails, is believed to recognize similar sites to PPCs [7]. Hence, the mechanism of proteolytic cleavage and the group of enzymes involved in cleavage varies between species, and also tissue types, and it is likely that many substrate-specific proteases are yet to be identified [4]. For this reason, algorithms designed to predict propeptide cleavage sites in all eukaryotes can fare poorly when used to predict propeptide cleavage sites in precursors of venom proteins due to differences in protease recognition patterns between species.

Propeptide cleavage in spider-toxin precursors follow the loose constraints of the Processing Quadruple Motif (PQM), where an arginine residue at position −1 partners a glutamic acid residue at either positions −2, −3 or −4 [8]. However, in certain species such as Australian funnel-web spiders, the motifs ‘KR’ and ‘RR’ at positions −2 and −1 are also used [9].

We evaluated several machine-learning strategies based on their ability to correctly identify propeptide cleavage sites in spider toxins. The final predictor we developed (SpiderP) is based on a linear support vector machine and is available through the ArachnoServer website [9]. To test the accuracy of our tool we used cross-validation and generated new experimental datasets. We used our tool to gain new biological insights into venom complexity by uncovering novel structurally variable toxins in the venom of an Australian tarantula.

SpiderP provides a simple interface that allows users to input amino acid sequence(s) in FASTA format (see http://www.arachnoserver.org/spiderP.html). The algorithm outputs a list of predicted precursor and mature peptide sequences, and it can easily be modified as more experimentally confirmed sites are identified. SpiderP incorporates SignalP [10] to predict signal peptides, which are removed prior to performing propeptide predictions (Fig. 1).

## Results and Discussion

### Training and Test Sets

**Figure 2:**
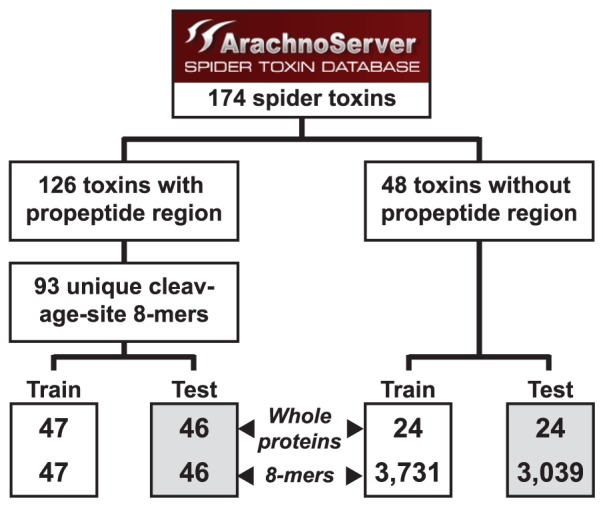
Partitioning of cleavage-site positive and cleavage-site negative training and test sets.

Starting with 126 spider toxin sequences, we created positive training and test sets for both whole proteins and 8-mers (Fig. 2). We began by assembling the set of 8-mers centered on the known cleavage site in each of the 126 toxin proteins. Following removal of duplicate 8-mers, the set comprises 93 distinct 8-mers. We then randomly split this set of 8-mers into approximately equal-sized training and test sets (47 and 46 8-mers, respectively) and created whole-protein training and test sets based on the 8-mer training and test sets. The whole-protein training set contains one representative protein for each of the 47 members of the 8-mer training set. Similarly, the test set contains 46 proteins, one for each member of the 8-mer test set. The positive whole proteins in the training and test sets are labeled with the position of the known cleavage site.

We also created negative sets of proteins and 8-mers. The negative set of proteins comprises 48 spider toxins that do not encode propeptides. Here, we began by first randomly splitting the 48 sequences into equal-sized whole-protein training and test sets (24 sequences each). We then created the 8-mer training and test sets consisting of the set of all 8-mers that occur in each of the corresponding whole-protein sets. The negative 8-mer training and test sets contain 3,731 and 3,039 cleavage-site negative 8-mers, respectively.

The 8-mer training set was only used to learn the user-settable parameters of each type of 8-mer classifier, as described below under each classifier heading. We then fixed those parameters to the learned values and assessed the accuracy of the 8-mer classifier using cross-validation on the test 8-mer test set. To create the whole protein classifier, we trained the 8-mer classifier on the entire training set of 8-mers. We then tested the resulting whole-protein classifier using the test set of whole proteins. All testing and training sets are available through http://www.arachnoserver.org/spiderP.html.

Given the small dataset and imbalance between positive and negative datasets, we used SVMs that assigned different misclassification costs to each class by assuming that the number of misclassified examples from each class is proportional to the number of examples in each class to address the problem of overfitting (see Methods). Also, cross-validation datasets were randomly assigned from positive and negative classes to minimize potential overfitting bias. Moreover, because of the skewed dataset we present precision-recall curves, in addition to receiver operating characteristic (ROC) curves. Precision-recall curves are able to provide a more insightful picture of an algorithm's performance when the number of negatives greatly exceeds the number of positives. Undoubtedly, the true distribution of toxins in nature would be more accurately represented by a larger positive randomly sampled dataset. However, at this time, the cost and time in obtaining the required experimentally validated data is prohibitive.

### Algorithm

We explored several propeptide cleavage site prediction strategies. Cleavage of propeptides is based on protease recognition of specific amino acid residues surrounding the site of cleavage, so our predictors predict whether each of the overlapping 8-mers in a protein contains a cleavage site between the fourth and fifth residue. Before evaluating 8-mers, however, our predictors first predict and remove any (putative) signal peptide from the N-terminal end of the protein. They then predict whether each overlapping 8-mer contains a cleavage site and choose the predicted cleavage site that is (i) closest to the most N-terminal cysteine residue in the protein and (ii) N-terminal to it. If the protein contains no cysteine residue, the classifier chooses the most C-terminal predicted cleavage site, based on current biological knowledge [9]. Given an input protein sequence, each of our propeptide cleavage site prediction strategies outputs a single predicted cleavage site position, or predicts that the protein does not contain a propeptide cleavage site.

To create classifiers for predicting whether a given 8-mer contains a propeptide cleavage site, we trialed three distinct machine learning strategies: support vector machines (SVMs) (linear and radial basis function (RBF) kernel), decision trees and hidden Markov models (HMMs). To predict and remove any (putative) signal peptide from the input protein, our propeptide cleavage site predictors used the SignalP 4.0 algorithm [10].

We trained SVM, Decision Tree and HMM classifiers to classify 8-mers as to whether they contain a cleavage site using the training set (Fig. 1). We then measured the accuracy of each 8-mer classifier (except HMM) on an independent test set of 8-mers using 10-fold cross-validation. From each 8-mer classifier, we also constructed propeptide cleavage site predictors as described above, and tested the ability of these predictors to exactly predict the correct cleavage site using the test-set proteins. The results for each type of classifier are shown below.

### SVM (linear)

**Figure 3:**
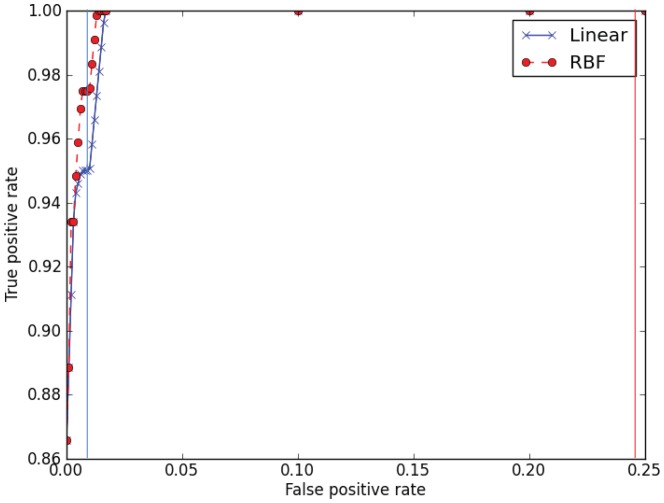
Cross-validated ROC curves for predicting if an 8-mer contains a cleavage site using linear (×) or RBF kernel (•) SVMs. Each point shows the average true positive rate (over the cross-validation folds) at the given FPR. Vertical lines denote the average false positive rate (1–specifity) (over cross-validation folds) when the SVM score threshold = 0 (the solid line corresponds to the linear SVM and the dashed line corresponds to the RBF SVM). Cross-validation was performed on the 8-mer test set.

SVMs have been widely used to identify the cleavage sites of protease substrates [11]. In this study, we use both linear and Gaussian RBF kernels. We first transformed our sequences using a method of binary encoding (described in Methods) and then used model selection on the 8-mer training set to obtain an overall soft margin value (*C*) of 1.0. Ten-fold cross-validation was then used to determine the predictive ability of this classifier on 8-mers. We observed an overall accuracy (see Methods for definitions of accuracy measures) of 98.9%, a balanced accuracy (taking into account uneven sample sizes) of 98.2%, a sensitivity score of 97.5%, a specificity score of 98.9%, and a false positive rate (FPR) of 0.01 at the default score threshold of 0, averaged over all folds (Table 1). Fig. 3 shows the ROC curve for predicting if an 8-mer contains a cleavage site, plotting the true positive rate versus the false positive rate for varying score thresholds. When we use the trained linear SVM in our whole protein prediction framework to predict the exact location of the cleavage site (or its absence), the sensitivity and specificity on the independent test set were 97.7% and 93.8%, respectively.

**Table 1:** Mean cross-validated test set accuracy (standard error) of 8-mer classifiers.

Classifier	Sensitivity (%)	Specificity (%)	False positive rate	Balanced accuracy (%)
SVM	97.5	98.9	0.011	98.2
(linear)	(2.5)^a^	(0.1)	(0.001)	(1.2)
SVM	100	97.5	0.0247	**98.8**
(RBF)	(0)	(0.36)	(0.0036)	(0.18)
Decision	61.5	99.7	0.0034	83.9
tree	(1.9)	(0.05)	(0.0005)	(0.97)

### SVM (RBF)

Using grid search on our 8-mer training set, we obtained a *C* value and γ parameter of 1000.0 and 0.1, respectively. As with the linear SVM, we used the default score threshold of 0 to classify 8-mers. The RBF classifier yielded a better balanced accuracy and sensitivity compared to the linear SVM on 8-mers (Table 1). The result is consistent with the ROC curve (Fig. 3), which suggests that the SVM with the RBF kernel has higher sensitivity (true positive rate) at all false positive rates (FPRs) than the SVM using the linear kernel. Nonetheless, the RBF SVM performs worse than the linear SVM when used in our whole protein prediction framework for predicting propeptide cleavage sites (Table 2). Despite the fact that the underlying 8-mer classifier has better balanced accuracy, the whole-protein cleavage site predictor based on the RBF SVM has lower sensitivity and lower specificity than the predictor based on the linear SVM (discussed below).

**Table 2:** Accuracy in predicting the exact location (or absence) of a cleavage site in whole proteins measured on an independent test set.

Underlying Classifier	Sensitivity (%)	Specificity (%)	Balanced accuracy (%)
SVM (linear)	95.5	100	**97.8**
SVM (RBF)	84.1	95.8	90.0
HMM	88.6	100	94.3

### Decision tree

We used our tree classifier to predict proteolytic sites on our 8-mer test set. On 8-mers, the method achieved a high level of cross-validated specificity (99.7%) but low cross-validated sensitivity (61.5%). Unlike SVMs, which have a score threshold, decision trees make ‘hard’ (e.g., binary) classifications; hence we cannot report ROC values. Because the performance of the trained classifier is already substandard compared to the other classifiers we trialed on 8-mer sequences (Table 1), we did not further test the algorithm with whole sequences.

### HMM

Against whole sequences in the independent test dataset, the HMM propeptide predictor produced 88.6% sensitivity and 100% specificity (Table 2). The HMM propeptide predictor is more sensitive and specific than the RBF SVM predictor but does not perform as well as the linear SVM on the test data. It has a balanced accuracy of 94.3% measured on an independent test set.

### SpiderP

**Figure 4:**
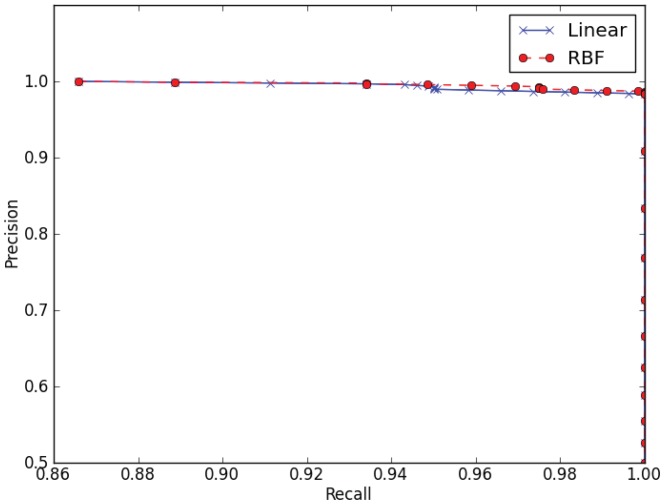
Cross-validated precision-recall curves for predicting if an 8-mer contains a cleavage site using linear (×) or RBF kernel (•) SVMs. Recall is equivalent to the TPR and the precision rate/positive predictive value is defined as TP/(TP+FP). Each point shows the average recall (over the cross-validation folds for a set FPR) versus the precision (the solid line corresponds to the linear SVM and the dashed line corresponds to the RBF SVM). Cross-validation was performed on the 8-mer test set.

Our results show that the whole protein predictor based on an SVM with a linear kernel is the most effective at predicting the location of propeptide cleavage sites in spider toxins (Table 2). This may seem surprising, given that the RBF kernel has better balanced accuracy for predicting if an 8-mer contains a cleavage site (Table 1) and that although precision-recall curves for both linear and RBF kernels show high precision for high recall rates, the RBF kernel performs slightly better than the linear kernel (Fig. 4). The reason for this is because the false positive rate of the linear kernel is much lower than that of the other 8-mer classifiers (Table 1 and Fig. 3, vertical lines). For a given false positive rate, as the length of the input sequence increases, the expected number of false positives will increase with sequence length following a cumulative binomial distribution. Because the propeptide predictor must predict the exact location of the cleavage site for a prediction to be counted as correct, it is important that the underlying 8-mer classifier makes few false positive predictions. In other words, if the 8-mer classifier has high a FPR, it is likely that the propeptide predictor will select the wrong putative cleavage site, or that a cleavage site will be predicted when there is none. The explanation for why the RBF kernel gives a much higher mean FPR (0.0247) when the kernel is a better performer than the linear kernel in the ROC curve is based on the fact that the final result is based on the default score threshold of zero, which yields a less than optimal score (See vertical lines in Fig. 3; the FDR for the linear kernel (blue line) is lower than the RBF FDR (red line) at the default score threshold.) Hence, it can be seen from Fig. 3 that it would, in principle, be possible to increase the score threshold of the RBF 8-mer classifier in order to lower its FPR, and it might be possible to find the optimum score threshold using the training set (in terms of balanced accuracy of the propeptide predictor); however, we did not explore this approach. We note that it is also possible that an integrated tool combining SVM with HMM may yield further increases in accuracy. SpiderP achieved 75% accuracy on independent test sequences that contain a propeptide but no cysteine framework.

To produce the final algorithm that is available on the ArachnoServer website (http://www.arachnoserver.org/SpiderP.html), we used the linear SVM 8-mer classifier. We used the *C*-value derived from the training set (*C* = 1.0, adjusted to take into account unbalanced examples) to train the model on the entire dataset. The final SVM 8-mer classifier contains 442 support vectors.

### Accuracy of SpiderP on new protein data

**Figure 5:**
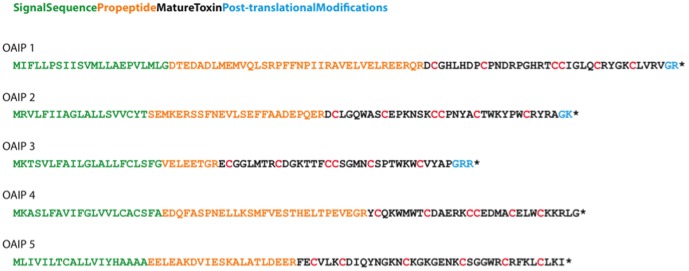
*Selenotypus plumipes* toxin sequences determined through Edman degradation. Signal peptides, propeptides, mature toxins and post-translational modifications are green, orange, black and blue, respectively. Cysteines resides in the mature toxin are in red.

To test the accuracy of the algorithm on new toxins with experimentally determined propeptide cleavage locations, we used Edman degradation to sequence five mature peptides from the venom of the Australian tarantula *Selenotypus plumipes* (Araneae: Mygalomorphae: Theraphosidae) (Fig. 5). The peptides comprise six cysteine residues that form three disulfide bonds in the mature toxin. The complete sequences of the prepropeptide precursors encoding these toxins were obtained by BLASTing the mature toxin sequences against a venom-gland transcriptome. This is the first time that spider-venom peptides have been sequenced from this spider genus, and the 8-mers containing the cleavage site in each of the five proteins are distinct from all cleavage site 8-mers in the training set. SpiderP correctly predicted 80% of propeptide cleavage sites in this small test set with an 8-mer specificity of 99.6% (1 FP).

### Comparison of SpiderP with other propeptide predictors

Existing programs for predicting propeptide cleavage sites showed low sensitivity and specificity on our dataset of 174 spider toxin precursors. ProP 1.0 [12] is based on neural networks that primarily identify the dibasic sequences RR and KR recognized by PPCs. On the set of known spider toxins used for this study, ProP yielded 12.2% sensitivity and 60.4% specificity. On our set of novel tarantula-venom peptides, ProP showed 0% sensitivity. Spiders use other enzymes in addition to PPCs and this probably accounts for the observed low accuracy. The ConoServer precursor sequence analysis tool [13], which was designed to identify propeptide cleavage sites in toxin precursors from marine cone snails, performs much better on the same spider-toxin dataset, with sensitivity and specificity values of 82.1% and 85.4%, respectively. On our novel set of tarantula-venom peptides, ConoServer showed 80% sensitivity. The ConoServer tool uses a rule-based, pattern-matching approach that takes into account arginine cleavage by glutamic acid control (E to R rule), a cleavage type that also exists in spiders but not in mammals [4], [8]. However, ConoServer is unable to predict propeptide cleavage sites in linear toxins that do not contain a cysteine framework, which represent a sizable fraction of our toxin dataset. This is unsurprising given that despite convergent evolution of some toxin genes, toxins have evolved independently in different lineages, and have adapted different strategies, complicating generalization across taxa. In contrast to cone snails and other eukaryotes, spiders do not appear to use “KK” or “RK” as propeptide cleavage sites.

### Predicting novel toxins from the venom gland transcriptome of an Australian tarantula

Using SpiderP and homology information, we uncovered and analyzed 970 unique mature-toxin sequences from the venom-gland transcriptome of the Australian tarantula *S. plumipes*. A putative toxin is defined by the presence of both predicted signal peptide and propeptide, either a high degree of similarity to known spider toxins, or the presence of four or more cysteines in the predicted mature toxin sequence. Due to possible sequencing and assembly errors the number of sequences we obtained is likely to be an overestimate of the actual number of toxin sequences. Thus, all sequences were clustered into groups based on three or more homologous contigs and similarity to known spider toxins were determined as described below. All reads were quality clipped and we note a high average consensus quality (MIRA: 33) and the absence of any unresolved repeat positions across contigs. Raw cDNA reads were assembled into contigs and six-frame translated into amino acid sequences (136,469 six-frame translated sequences). We considered contigs as putative toxins if both signal sequences and propeptides were predicted. 3,514 sequences met these criteria. Of these, 2,761 were highly similar to known spider toxins (E-value<10^−5^). Given that spider toxins tend to be cysteine-rich, we also kept sequences (149) exceeding this E-value threshold if four or more cysteines were present in the predicted mature toxin sequence.

**Figure 6:**
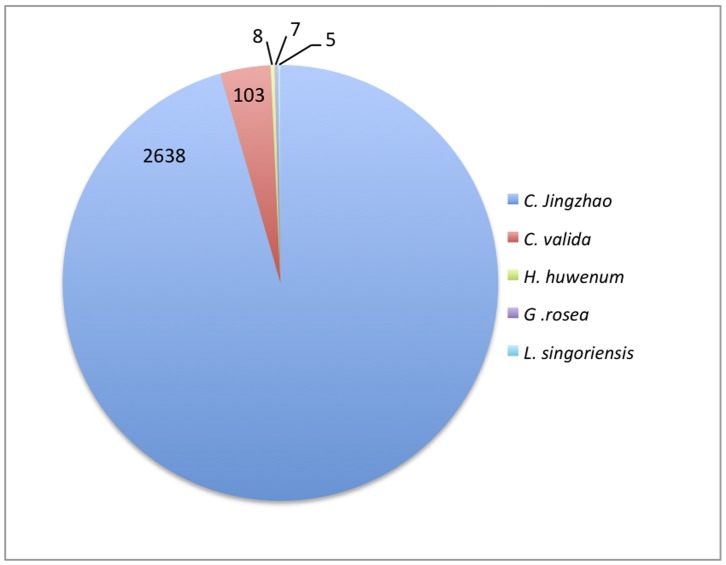
Pie chart showing the relative number of top BLAST hits from *Selenotypus plumipes* sequences to toxins of different species from the ArachnoServer database. Numbers represent the counts of *S.plumipes* sequences that match known toxins from species, including *Chilobrachys jingzhao* (Chinese earth tiger tarantula), *Coremiocnemis valida* (Singapore brown tarantula), *Haplopelma huwenum* (Chinese bird spider), *Grammostola rosea* (Chilean rose tarantula), and *Lycosa singoriensis* (wolf spider). With the exception of *L.singoriensi*s, all of these spiders are primitive mygalomorphs that are closely related to *S. plumipe*s.

The vast majority of sequences (2,638) matched 29 toxins from eight classes of toxins of unknown molecular target and function with inferred ion channel modulating activity from the Chinese earth tiger tarantula, *Chilobrachys jingzhao* (Fig. 6, Dataset S1) [14]. A small fraction of the *S. plumipes* sequences were most similar to toxins from other tarantula species, not *C. jingzhao*. These toxins included a *Coremiocnemis valida* insecticidal toxin (UniProt accession number P82601) [15], *Haplopelma huwenum* toxins with putative trypsin inhibitory and voltage-gated potassium channel blocking activities (UniProt B2ZBB6 and B3FIVO), and a *Grammostola rosea* toxin that targets mechanosensitive channels (UniProt Q7YT39) [16]. Four peptides were most similar to a CAP (cysteine-rich secretory proteins, antigen 5, and pathogenesis-related 1 proteins) domain-containing venom peptide from *Lycosa singoriensis* [17] (UniProt A9QQ26, E-value<10^−7^). This is a distantly related araneomorph species that last shared a common ancestor with the mygalomorph *S. plumipes* more than 250 million years ago. The sequences from the two species only share slight similarities in their N-terminal regions but are highly divergent at their mature C-termini indicating that the similarities are coincidental and not reflective of true evolutionary history (Fig. S1). Notable differences between the *S. plumipes* and *L. singoriensis* peptides include the absence of a propeptide in the *L. singoriensis* precursor compared with its predicted existence in *S. plumipes*, and fewer cysteine residues (2–7) in the *S. plumipes* toxins compared with the peptide from *L. singoriensis* (19).

**Figure 7:**
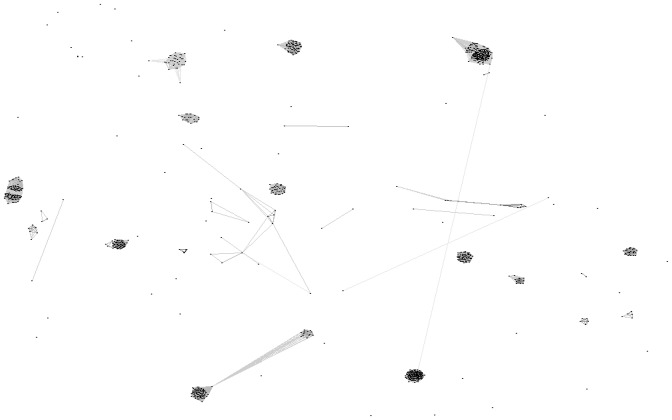
Representation of a 3D image from the clustering of predicted *S.* *plumipes* toxin sequences by homology showing distinct clusters. A sequence is represented by a single dot. Connecting lines indicate a BLAST similarity where p<10^−15^.

Given the large number of putative toxins found, we clustered all predicted mature toxins from *S. plumipes* to approximate functional groupings based on homology. 29 groups were identified containing clusters of two sequences or more with 85 sequences that did not cluster (Fig. 7, Dataset S2). There is high jackknife support for all groups (at least 89%). The average identity within groups is 70%, where *n*>3. Our data supports the expression of at least 29 distinct classes of toxins in *S. plumipes*. Each of these groups may comprise closely related gene family members and/or different allelic variants. To err on the side of caution, only groups containing three or more sequences were considered a functional class and further analysed.

To describe novel groups we used BLAST to search all grouped sequences, where the number of sequences in a clade (*n*) is >5, against a database of signal peptides from ArachnoServer. Signal peptides are conventionally used to assign toxins into ‘superfamily’ groupings [3], [18], as toxins tend to evolve rapidly in mature and propeptide regions. We found seven out of 17 groups of toxins (where *n*>5) that did not match to known spider toxin signal peptides using an E-value cut-off of 10^−5^ (groups D, E, H, L, M, N and O). Four of these groups (D, M, N and O) did not match any sequences even at an E-value cut-off of 1, indicating that these are novel categories of toxin sequences. Whether these groups of toxins are specific to the *S. plumipes* lineage will require future transcriptomic analysis of related species.

**Figure 8:**
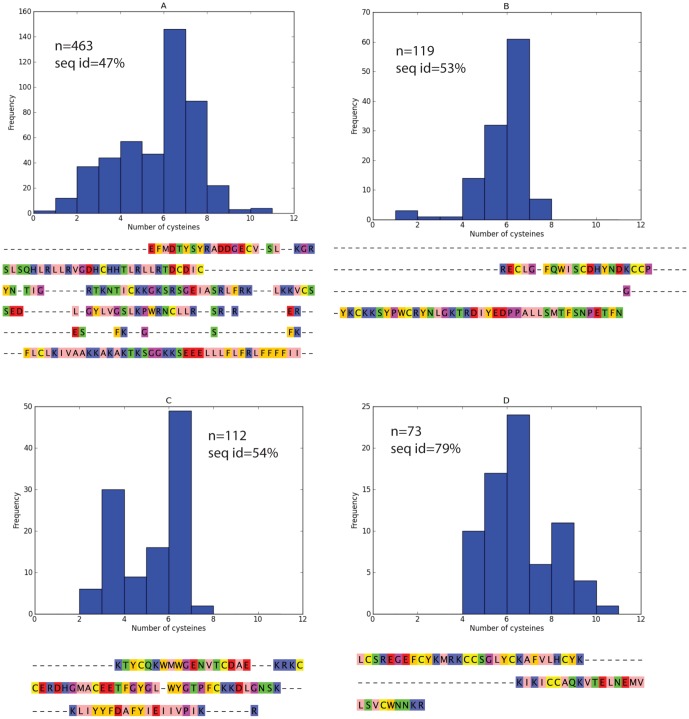
For groups clustered by CLANS, a histogram of cysteine numbers in the mature peptide with the consensus sequence below. For each of the 29 groups clustered by CLANS based on homology, we assigned labels A to Q to the groups (based on the total number of sequences within each group ordered from largest to smallest). The consensus sequence represents the most frequent residue in each column of the multiple sequence alignment. A dash ‘−’ denotes cases where the most frequent character is empty at an alignment position. Groups A to D are presented in this figure (For analogous information for other sequence groups see Figures S2, S3, S4). ‘n’ denotes the number sequences in the group. The percent sequence identity for each group (‘seq id’) is shown.

Due to their role in the formation of disulfide bonds, the number of cysteines plays a critical role in determining the structure and function of venom proteins. The number of encoded cysteines in the predicted *S. plumipes* toxins varied from zero to 16. As expected, the most frequent number of cysteines is six, the number required to form the highly stable inhibitor cystine knot (ICK) structural motif [19], which is common in spider-venom peptides [20]. The ICK motif comprises a ring formed by two disulfide bonds and the intervening sections of polypeptide backbone, which is bisected by a third disulfide. Peptides containing the ICK motif can function as ion channel blockers, antimicrobial and antiviral molecules, or as haemolytic agents [21]. Surprisingly, *S. plumipes* toxin cysteine numbers varied dramatically within homology groups. We identified variation in cysteine numbers and sequence lengths within each group (*n*>3) along different toxin lineages (Fig. 8, Fig. S2, S3, S4). As the ICK structure promotes protein folding and is highly thermodynamically stable, we expected the number and spacing of cysteines to remain relatively invariant within a group, being subjected to a high level of purifying selection. Our result points to diversifying selective pressure in promoting the addition and deletion of cysteines to facilitate novel structure and function in *S. plumipes* toxins. We note that incomplete sequencing and sequencing errors that cause premature translation termination will cause artifactual variations in sequence length. However, many of short sequences are highly homologous, suggesting that the observed variants are unlikely to be sequencing artifacts (Dataset S2). Integration of proteomic results, including confirmation of these putative sequences by peptide mass matching, together with functional characterization of these toxins, and comparative analyses of venoms between spider species will help to understand the genetic basis underlying the diversity of spider venom proteomes and are challenges for future research.

### Summary

Constant advances in sequencing technologies are yielding an unprecedented amount of DNA and RNA sequence data. In the face of this deluge of sequence data, an automated system is necessary to extract the active portion of venom proteins for further study. We have developed a strategy that meets this need. Our model employs an SVM algorithm and not only takes into account residues preceding the cleavage site but also incorporates trailing residues, which are also likely to play a role in substrate recognition. Our program shows high sensitivity, and can distinguish, with high accuracy, propeptide-containing toxins from those that do not encode prodomains. Importantly, it also performs well on toxin sequences that do not contain a cysteine scaffold.

## Methods

### Measuring accuracy

Balanced accuracy is defined as [sensitivity+specificity]/2, where sensitivity = TP/[TP+FN], specificity = TN/[TN+FP], and where TP = number of true positives, FP = number of false positives, TN = number of true negatives, FN = number of false negatives. Overall accuracy is defined as [TP+TN]/[TP+FN+FP+TN]. False positive rate is defined as 1–specificity. Precision rate/positive predictive value is defined as TP/[TP+FP].

### Support Vector Machines

To encode amino acid residues for SVM input, we chose the simpler unary residue-encoding scheme over PSI-BLAST position-specific scoring [22]. For protease cleavage site prediction in HIV sequences, simple linear classifiers are as effective as more complex models suggesting that more sophisticated strategies may be redundant [23]. Hence, we transformed 8-mer peptides into 161-dimensional input vectors for our SVMs. First, each amino acid is encoded by a 20-dimensional vector made up of zeros and ones, where positions in the vector correspond to the 20 possible amino acids in alphabetical order according to their standard one-letter abbreviations. For example, alanine is represented by [1,0,0,0,0,0,0,0,0,0,0,0,0,0,0,0,0,0,0,0], cysteine by [0,1,0,0,0,0,0,0,0,0,0,0,0,0,0,0,0,0,0,0], etc. Concatenating the appropriate 20-dimensional encodings of the 8 residues gives a 160-dimensional vector. Second, in order to encapsulate positional information with respect to the location of the 8-mer along the protein sequence, we linearly scaled the location of the 8-mer to values in the interval [0,1], with 0 indicating that the particular 8-mer was extracted from the N-terminus of the protein. Concatenating this scalar with the 160-dimensional vector encoding the 8-mer residues yields a 161-dimensional vector.

We used the Python PyML package (http://pyml.sourceforge.net) to construct the SVMs. We use cross-validation on a training set of 8-mers to determine the soft-margin constant (*C*) and the γ parameter for the Gaussian RBF (see below for descriptions of training set creation), iterating over a spectrum of values for *C* (0.1, 1, 10, 100, 1000) and γ (0.01, 0.1, 1, 10). We set the values of C and γ to those that maximized the cross-validated balanced accuracy. Due to the relative rarity of proteolytic sites, there are considerably more negative than positive instances. To address this dataset imbalance, we use a feature of the PyML machine learning package that increases the penalty factor associated with the misclassification of the positive class [24]. The total misclassification cost (SVM soft-margin constant, *C*) is split into to terms that assign different values of the soft-margin constant for the positive (*C*_+_) and negative *C*_−_ class, such that *C*_+_ *n*_+_ = *C*_−_ *n*_−_, where *n*_+_ and *n*_−_ are the numbers of positive and negative examples, respectively [24].

### Decision Trees

We used a decision tree classification algorithm implemented in the MATLAB Statistics Toolbox (R2010a; MathWorks, Natick, MA, USA) to construct a predictor for propeptide cleavage. We used a form of vector-based encoding similar to the SVM input, but excluding positional information. We determined the pruning depth for the tree using cross-validation on the 8-mer training set, iterating over five pruning depths defined by the MATLAB function ‘prune’, which is based on an optimal pruning scheme that successively prunes grouped branches that give the least improvement in error cost.

### Hidden Markov Models

Although the HMMER package is widely used to construct profile hidden Markov models (profile HMMs) for sensitive database searching, it has also been successfully used to build profile HMMs for eukaryotic signal peptide detection [25]. We used HMMER 3.0 (www.hmmer.org) to construct an HMM for the positive 8-mer training set using amino acid sequences in the form of a FASTA file. From these sequences, we used the HMMER command ‘hmmbuild’ to construct an HMM. We then used the command ‘hmmpress’ to prepare the HMM database for searching of whole sequences for which we used the command ‘hmmscan’. For maximal sensitivity, we used the ‘–max’ parameter to turn off speed-increasing filters to include low scoring hits.

To predict the cleavage site, we used E-values assigned by the program ‘hmmscan’ to portions of the whole protein sequence matching the HMM profile. For each reported alignment, we checked whether the conditional E-value is less than 1 and inferred cleavage sites to only occur subsequent to arginine residues [9]. We then only keep alignments where arginine residues are N-terminal to any cysteines within the whole protein (minus the signal peptide). Where multiple alignments are identified, the arginine residue contained within the alignment with the lowest E-value is selected as the residue immediately N-terminal to the point of propeptide cleavage.

### N-terminal sequencing of five crude venom peptides

A single milking of an adult *S. plumipes* adult (∼10 g total body weight) yielded 351±9 mg dry weight/mL of venom (average of four fortnightly milkings of 10 individuals). Venom was fractionated by reverse-phase (RP) HPLC using a Vydac C18 analytical RP-HPLC column (Grace Division, Deerfield, IL). Solvent A was 0.1% trifluoroacetic acid (TFA) in water and Solvent B was 0.1% TFA in acetonitrile. Venom was eluted at a flow rate of 1.0 mL/min, yielding ∼50 peaks that eluted before 60% Solvent B. We selected RP-HPLC fractions with insecticidal activity (determined by injection into mealworms) and further fractionated these fractions using an orthogonal cation exchange chromatography step in order to isolate individual insecticidal peptides, which were then desalted using RP-HPLC. Free cysteines in the peptides were then alkylated using 4-vinylpyridine. Alkylated samples were then sent to the Adelaide Proteomics Centre (Adelaide, South Australia) and the Australian Proteome Analysis Facility (Sydney, New South Wales) for N-terminal sequencing via Edman degradation.

### cDNA library preparation and transcriptome sequencing using Roche 454 GS-FLX platform

Four venom glands from two *S. plumipes* spiders were prepared and total RNA was extracted using Trizol. RNA concentration and quality was measured using a Nanodrop (ND-1000, ThermoScientific, Wilmington, DE, USA) and Bioanalyzer (Bioanalyzer 2100, Agilent Technologies, Santa Clara, CA, USA). An Oligotex Direct mRNA Mini Kit (Qiagen) was used to isolate poly A^+^ mRNA from the total RNA. Elution was performed first in 5 mM Tris-HCl (pH 7.5), and subsequently samples were precipitated with RNAse-free glycogen, sodium acetate, and ethanol. Samples were again resuspended in RNAse-free water, and then the RNA concentration and quality were measured using the Nanodrop and Bioanalyzer. A total of 227 ng of mRNA was submitted to the Brisbane node of the Australian Genome Research Facility for sequencing using the Roche 454 GS-FLX platform.

### Transcriptomic analysis

Sequences were assembled using MIRA version 3.4.1 using the parameters mrs = 99, egp = 1 and mrpc = 1 [26]. Thus, in order to be considered for assembly, the minimum percentage of matching between two reads has to be 99% (mrs = 99). The large penalty for alignments containing long gaps (egp = 1) was used to reduce erroneous long contigs. Sequences were quality-clipped prior to assembly. We were not stringent, however, with the minimum number of reads per contig required for assembly (mrpc = 1). This enabled us to capture potential weakly expressed genes with the view that downstream proteomics strategies will be capable of validating these sequences. As we aimed to define a high-level view of transcriptomic complexity by grouping homologous sequences by clustering (*n*>3), if sequencing and assembly errors are small and distributed randomly, these are unlikely to influence the total number of groups recovered. ‘Padded’ assembled reads were used (contigs for which there was minor evidence for additional bases). Six-frame translated sequences greater than 35 amino acids long and starting with a methionine were used as input into SpiderP. We used BlastP 2.2.24 [27] to search against sequences in the ArachnoServer database using an E-value cut-off of 10^−5^. CLANS [28] was used to cluster the sequences based on a P-value threshold of 10^−15^, and results were recorded after 570 successive rounds of iteration. Jackknife support was calculated after 100 rounds of resampling with 0.1% removal of data for resampling. Percent identities were calculated using MEGA5 [29]. The Poisson model and pairwise deletion were used. Multiple sequences were aligned using Muscle [30], and consensus sequences were calculated using Jalview [31]. Signal peptide searches were conducted using BlastP with an E-value cut-off of 10^−5^.

## Supporting Information

Figure S1 **Alignment of** ***L. singoriensis*** **toxin (UniProt A9QQ26) with** ***S. plumipes*** **sequences.** Gaps are denoted by dashes.
(EPS)

Figure S2 For CLANS clustered groups E to H, a histogram showing cysteine numbers in the mature peptide and the consensus sequence based on an alignment of all the mature peptide sequences in the group below. The consensus sequence represents the most frequent residue in each column of the alignment excluding gaps. ‘n’ denotes the number sequences in the group. The percent sequence identity for each group (‘seq id’) is shown.
(EPS)

Figure S3 For CLANS clustered groups I to L, a histogram showing cysteine numbers in the mature peptide and the consensus sequence based on an alignment of all the mature peptide sequences in the group below. The consensus sequence represents the most frequent residue in each column of the alignment excluding gaps. ‘n’ denotes the number sequences in the group. The percent sequence identity for each group (‘seq id’) is shown.
(EPS)

Figure S4 For CLANS clustered groups M to Q, a histogram showing cysteine numbers in the mature peptide and the consensus sequence based on an alignment of all the mature peptide sequences in the group below. The consensus sequence represents the most frequent residue in each column of the alignment excluding gaps. ‘n’ denotes the number sequences in the group. The percent sequence identity for each group (‘seq id’) is shown.
(EPS)

Dataset S1 Zipped file containing (1) All FASTA sequences of translated open reading frame contigs containing predicted signal and pro- peptides; (2) Parsed BLAST result of these sequences against the ArachnoServer database; (3) Parsed BLAST result of grouped sequences against signal peptides of known spider toxins.
(ZIP)

Dataset S2 **Zipped file containing CLANS clusters of sequences by similarity in FASTA format.**
(ZIP)
